# Artificial Respiration in New-born Children

**Published:** 1881-05

**Authors:** 


### ARTIFICIAL RESPIRATION IN NEW-BORN CHILDREN.

THE following is an abstract of the paper, entitled "On Artificial Respiration in New-born Children; the amount of ventilation secured by different methods; an experimental inquiry;" by Francis Henry Champneys, M. B. Oxon, M. R. C. P. The number of bodies experimented on was twenty-six, of which twenty were utilized for this part of the subject; only such as had never breathed being used. Tracheotomy having been performed, a cannula was tied into the trachea, the cannula being connected by an India-rubber tube, with a V tube filled with water, which thus registered inspiration and expiration by the rise and fall of the water, the results in the same body only being compared, and the highest effect being the standard of comparison. The methods used were nine—viz., that of Marshall Hall, Howard, Silvester, Pacini, Bain, Schücking, Schüller, Schroeder, Schultze. The conclusions were the following:—(1) Since the position of equilibrium of a still-born child's chest is one of absolute expiration, airlessness, or collapse, no method which depends on elastic recoil of the chest-walls will introduce air into its lungs. The methods of Marshall Hall and Howard are useless as means of directly ventilating the lungs of still-born children. (2) Silvester's method, and its modifications by Pacini and Bain, introduce more air into the lungs than any other method. (3) In using Silvester's method the arms should be held above the elbows and everted. (4) In using Pacini's or Bain's method the legs should be fixed, the second half of Pacini's method and Bain's second method should not be employed, as the weight of a new-born child's body is insufficient counterpoise to the necessary traction. (5) In using the two latter methods the operator may face the subject, and lift the shoulders from below; by this means he is able to watch the child's countenance, and is able to introduce an equal quantity of air. (6) Schücking's method is no improvement on Silvester's. (7) Schüller's method is useless, and not free from risk. (8) Schroeder's method is useless. (9) Schultze's plan, although its power of ventilation is less than that of Silvester and its modifications, yet acts efficiently. (10) In Schultze's method the diaphragm does descend, though but slightly; its principal action, however, is on the thoracic walls, as in the Silvester group. (11) In Schultze's method it is important that the whole weight should rest (at the end of the inspiratory movement) on the index-fingers in the axillæ, and should not be distributed to the other fingers. (12) The violence of the action of the method of Schultze is not in its favor. (13) Opisthotonos always produced expiration by tension of the anterior body walls, and should be avoided. Behm's experiments, six in number, dealt with children truly still-born in two cases only (one other having failed). His general conclusions speak highly of Marshall Hall's and Howard's methods, which, however, in the above two cases, were nearly or quite failures. His error is in not appreciating the collapsed state of the thorax in truly still-born children.—The President invited discussion from those who had had practical experience of these methods. He asked how long after birth was a still-born child recoverable; and also whether evils do not often attend the more violent measures, such as succussion or slapping, so often adopted.—Dr. Matthews Duncan said the paper was one of great value, which would lead to results of cardinal importance in the treatment of the still-born. Schultze's method appeared too violent, and he felt that it was desirable to induce respiration by as gentle means as possible. The author's remarks upon Howard's and M. Hall's methods showed these methods to be without practical importance or utility. His own experience was that the older methods, which he was taught were of great value, were conducted with objectionable haste and hurry, as well as violence. He remembered a case where the spleen and liver were ruptured by the over-active treatment employed in resuscitation. The method of direct inflation was unsatisfactory, the air being as often passed into the œsophagus and stomach as into the lungs. He was of the opinion that Silvester's method was the best. He pointed out that recovery was possible even if the heart had ceased beating for more than a minute. In such cases the heart was often temporarily arrested; so also in still-births.—Dr. Roper urged that the child frequently commenced to breathe spontaneously, even when artificial methods were being employed; e. g., he had seen a child inspire whilst the movement of expiration was being made by Silvester's method. The cessation of the cardiac beat was difficult to make out in breech cases, and he related three cases in which the child was left for dead. One of these occurred in the practice of the late Mr. Brown, of St. Mary Axe; the child was born still in the absence of the medical man; it was taken to the surgery and thence to the late Mr. Solly, who, next day, in dissecting the body, found that the heart was still beating. A second instance was of a fœtus of five months and a half, which was set aside for dead, Dr. Roper attending to the mother, who was suffering from hæmorrhage. He was astonished next day to find that this immature child, which had lain on the floor for eleven hours through a cold night, was breathing, and its heart beating. Again he once delivered a monster by the forceps; it seemed dead, and was not further attended to, but as he was leaving the room two hours later the child, which had been left aside, began to cry. Such examples showed that the new-born have greater tenacity of life than is supposed.—*London Lancet.*

